# The experimental heating of rye, oat, spelt, wheat and barley between 215 and 300 °C: the stable carbon and nitrogen isotope data and the photographic evidence of changes to the morphology of the grains

**DOI:** 10.1016/j.dib.2023.109544

**Published:** 2023-09-09

**Authors:** Elizabeth Stroud, Michael Charles, Amy Bogaard, Erika Nitsch, Helena Hamerow

**Affiliations:** a School of Archaeology, University of Oxford, UK; b Cabbage Moor, Great Shelford, Cambridge CB22 5NB, UK

**Keywords:** Stable isotope analysis, Charring offset, Temperature and duration heating experiment, Archaeology, Archaeobotany

## Abstract

The effect that heating has on cereal grain morphology and isotopic values has far reaching consequences for archaeobotanical research and palaeodietary reconstructions. Stable carbon and nitrogen isotopic data and mass loss percentages on, and photographs of, rye, oat, barley, wheat and spelt from a heating experiment are presented and support Stroud et al. (2023). The experiment heated rye, oat, and spelt at 215 °C, 230 °C, 245 °C, 260 °C and 300 °C for 4 h, 8h and 24 h, with each temperature/duration condition consisting of 3 samples of 10 grains per sample. The mass loss of the grains, the %C and %N, and *δ*^13^C and *δ*^15^N values are presented. Furthermore, photographs of the grains’ external and internal morphology for each temperature/duration combination are provided. The wheat and barley data of samples charred between 215 °C and 260 °C/ 4–24 h were obtained from the published and unpublished dataset of Nitsch et al. (2015) and it is this dataset which the new data builds upon. This article also provides the published and unpublished data and photographs from Nitsch et al. (2015), bringing together a dataset of nine crop species. This article provides the raw data from two cereal grain heating experiment, which will enable further research into understanding the impact of heating on both grain isotopic values and grain morphology. It also allows users to construct charred-uncharred isotopic offsets for a combination of species relevant to their research.

Specifications Table

**Table:** 

Subject	Social Sciences - Archaeology
Specific subject area	Stable carbon and nitrogen isotope analysis Grain morphology Charring and heating Cereals
Type of data	Tables Csv files Images Graphs R script
How the data were acquired	Wheat, rye, oat, barley and spelt were experimentally charred in a Gallenkamp Plus II oven at a range of temperatures and time durations (215–300 °C, 4–24 h). The samples were weighed before and after heating to obtain the mass loss percentage. The samples were analysed on a Sercon EA-GSL mass spectrometer at the University of Oxford's Research Laboratory for Archaeology and the History of Art to obtain stable carbon and nitrogen isotope values. Microscopy of the experimentally heated grains was conducted using a Leica microscope with Lumenera camera. See [2] for details on einkorn, emmer, pea, and lentil charring.
Data format	Raw Analysed
Description of data collection	The new isotopic analysis of the experimentally heated cereal grains was conducted using separate carbon and nitrogen runs due to the limited %N of some species. An internal alanine standard was used to drift correct the isotopic data, while a two-point normalisation was conducted on the majority of samples using IAEA-N1 and IAEA-N2 (nitrogen) and IAEA-C6 and IAEA-C7 (carbon). A small portion of rerun samples were normalised using an internal SEAL standard (seal collagen) and EMA-P2. Grains of each time/temperature combination were examined under a microscope and photographed. All graphing and statistical analysis were performed using R.
Data source location	Institution: University of Oxford City/Town/Region: Oxford Country: United Kingdom
Data accessibility	Repository name: Zenodo Direct URL to data: https://doi.org/10.5281/zenodo.8192962 DOI: 10.5281/zenodo.8192962
Related research article	E. Stroud, M. Charles, A. Bogaard, H. Hamerow, Turning up the heat: Assessing the impact of charring regime on the morphology and stable isotopic values of cereal grains. J. Archaeol. Sci., 152, (2023). https://doi.org/10.1016/j.jas.2023.105754. [1]

## 1 Value of the Data

• The data provided can be used to understand the impact of different combinations of heating temperature and duration on the *δ*^13^C and *δ*^15^N values of wheat, barley, rye, spelt and oat, as well as mass loss, %C and %N of the grains and changes to grain morphology
• Understanding the impact of heating on cereal grains’ *δ*^13^C and *δ*^15^N values is vital for archaeologists using such data for palaeodietary analysis and comparison with modern experiments. Furthermore, understanding the impact of heating on the morphology of different cereal species is of importance in archaeobotanical research, providing an understanding of the impact of heating on identification features, as well as taphonomic consideration such as survivability
• The presented data allow for the construction of isotopic offsets between the charred and uncharred archaeobotanical material which is important when comparing with uncharred material or when the isotopic values are used for palaeodietary reconstruction
• Photographs of the heated grains provide a library against which archaeological grains can be compared, allowing for the selection of suitable grains for isotopic analysis, as well as research into the effect heating has on grain morphology
• The data allows other archaeologists to tailor their understanding of the impact of heating to their specific crop suite and can be used with data from Nitsch et al. [2] to construct sites specific charring offsets.

## 2 Objective

Understanding the impact of charring on plant remains is vital for archaeobotanists, in particular those conducting isotopic analysis, as charring changes the morphology and isotopic value of seeds. Accordingly, data was generated to investigate the impact that charring at different durations and temperatures had on the morphology and isotopic values of cereal species which had not previously been examined (rye, oat and spelt). The newly generated data, which also includes increased temperature maximums for bread wheat and hulled barley, as well as previous data from the Nitsch et al. [2] study is presented below, providing a large corpus of isotopic data and images. Stroud et al. [1] provides in-depth analysis, morphologically and isotopically, of a subset of data presented here (rye, bread wheat, hulled barley and oat). The statistical analysis conducted in Stroud et al. [1], including the methods used to create the isotopic charring offsets is provided here as an R script to allow further investigation in to the impact of charring on different crop suites.

## 3 Data Description

This publication presents the stable carbon and nitrogen isotopic data of experimentally charred cereal grains; rye (*Secale cereale* L.), oat (*Avena sativa* L.), spelt (*Triticum aestivum* subsp*. spelta (*L.) Thell), bread wheat (*Triticum aestivum* L.) and hulled barley (*Hordeum vulgare* var. *distichum* L.), charred at 16 different temperature and duration combinations (215–300 °C 4–24 h). Additionally, the dataset from Nitsch et al.’s [2] charring experiment of 13 different temperature and duration combination (215–260 °C 4–24 h) is included of 5 species: bread wheat, hulled barley (husked and dehusked), einkorn (*Triticum monococcum* L.), emmer (*Triticum turgidum* subsp. *dicoccum* (Schrank)Thell*.),* pea (*Pisum sativum* L.), lentil (*Lens culinaris* Medik.).

Photographs showing the internal and external morphology of the experimentally charred items at the different temperature/time combinations are included. All data are stored on Zenodo with links included in the paper. Dataset 1 and Dataset 2 can be found here. A subset of these data have been used in Stroud et al. [1] to understand grain morphological changes which occur during charring as well as the isotopic differences between the different time and temperature combinations.

Tables 1 to 4 provide the analytical conditions of the isotopic measurements of all the newly conducted analyses and can be found here. Tables 1 and 2 provide the *δ*^13^C and *δ*^15^N values respectively, of the standards (both check and calibration) for every analytical session. Tables 3 and 4 provide the *δ*^13^C and *δ*^15^N values of the replicated samples used to understand the precision of the isotopic values.

Different formulae were used to understand accuracy, precision and standard uncertainty due to the combination of primary and secondary data. Table 5 shows the accuracy, precision and overall uncertainty of the data as per Szpak et al. [3] as well as the overall uncertainty for the combined Nitsch et al. [2] dataset and new data (as per [4]), and an overall uncertainty for the new data and selected wheat and barley data from Nitsch et al. [2] used in Stroud et al. [1]. The primary data were analysed following the protocol described in Szpak et al. [3] to calculate accuracy, precision and standard uncertainty. Due to the different methods used in the secondary data collection [2], accuracy, precision and standard uncertainty could not be calculated following Szpak et al. [3] due to the lack of check standards and sample duplicates. As such the Kragten [4] approximation method was used to calculated measurement uncertainties for each sample based on within run variability of the reference materials and internal standards, and the known uncertainties of the reference materials [5]. This was conducted on all data, as well as the subset used in Stroud et al. [1]: all new data and just hulled barley and bread wheat data from Nitsch et al. [2] were used in Stroud et al. [1] to characterise the species commonly found in early Medieval assemblages.

**Table 5:** The accuracy, precision and standard uncertainty of the new isotopic data as per Szpak et al. [1] as well as all data as per Kragten [4], and just the data used in Stroud et al. [1].

		*δ*^13^C_(VPDB)_ (‰)	*δ*^15^N_(AIR)_(‰)
Primary data	Accuracy (u(bias))	0.16	0.517
	Precision (u(Rw))	0.082	0.268
	Standard Uncertainty (Uc)	0.179	0.583
Primary and secondary data	Average measurement uncertainty	0.073	0.298
Early medieval crop suite used in Stroud et al. [1] (hulled barley, bread wheat, rye, and oat)	Average measurement uncertainty	0.077	0.309

Abbreviations used for the identification of charred material is as follows for the new dataset (labelled EAS in Author column of Dataset 1); the first two or three letters denote the species charred: hulled barley (BAR), bread wheat (FT), spelt (SPT), oat (OAT) and rye (RYE). The following three numbers are the charring temperature and fourth number is the duration. The final letter is the replication letter. Such information is also displayed separately within the dataset under the columns: Taxon, Time (hours), Temp (°C) and Replicate. The secondary data (with a EK in the Author column of Dataset 1) are abbreviated in a similar way in the ID column following the format that Nitsch et al. [2] used. Note that there is a hyphen between the charring temperature and batch number for the secondary data, and that HBH is used to indicate Hulled barley. The secondary data from Nitsch et al. [2] in Dataset 1 also includes the experimental charring of hulled and pearled barley batches (uncharred and charred at 230 °C for 24 h). Such data are included for completeness but not used in any of the graphs below, nor in Stroud et al. [1].

**Fig. 1:**
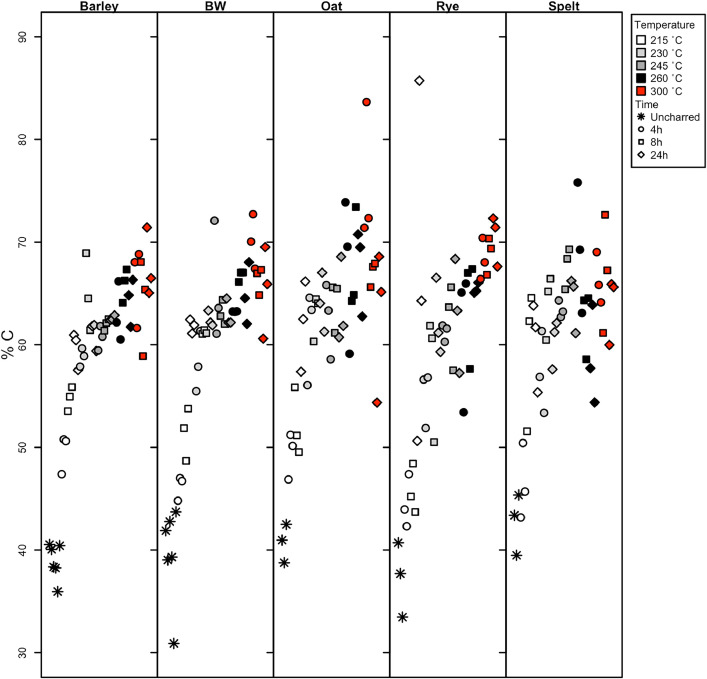
The %C of the five species charred up to 300 °C showing the changes in %C from uncharred through to 300 °C for 24 h (barley = hulled barley, BW = bread wheat).

**Fig. 2:**
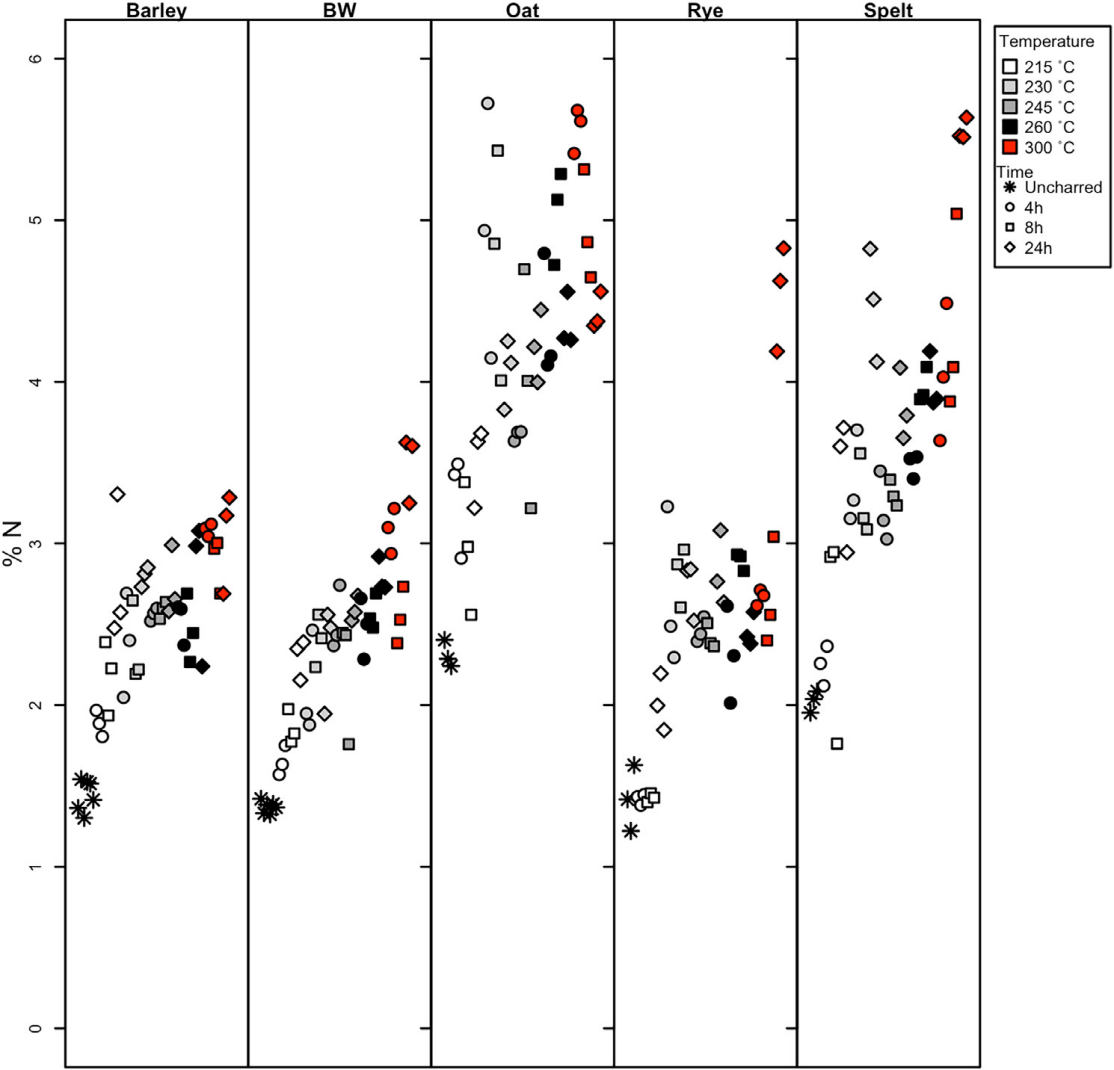
The %N of the five species charred up to 300 °C showing the changes in %N from uncharred through to 300 °C for 24 h (barley = hulled barley, BW = bread wheat).

**Fig. 3:**
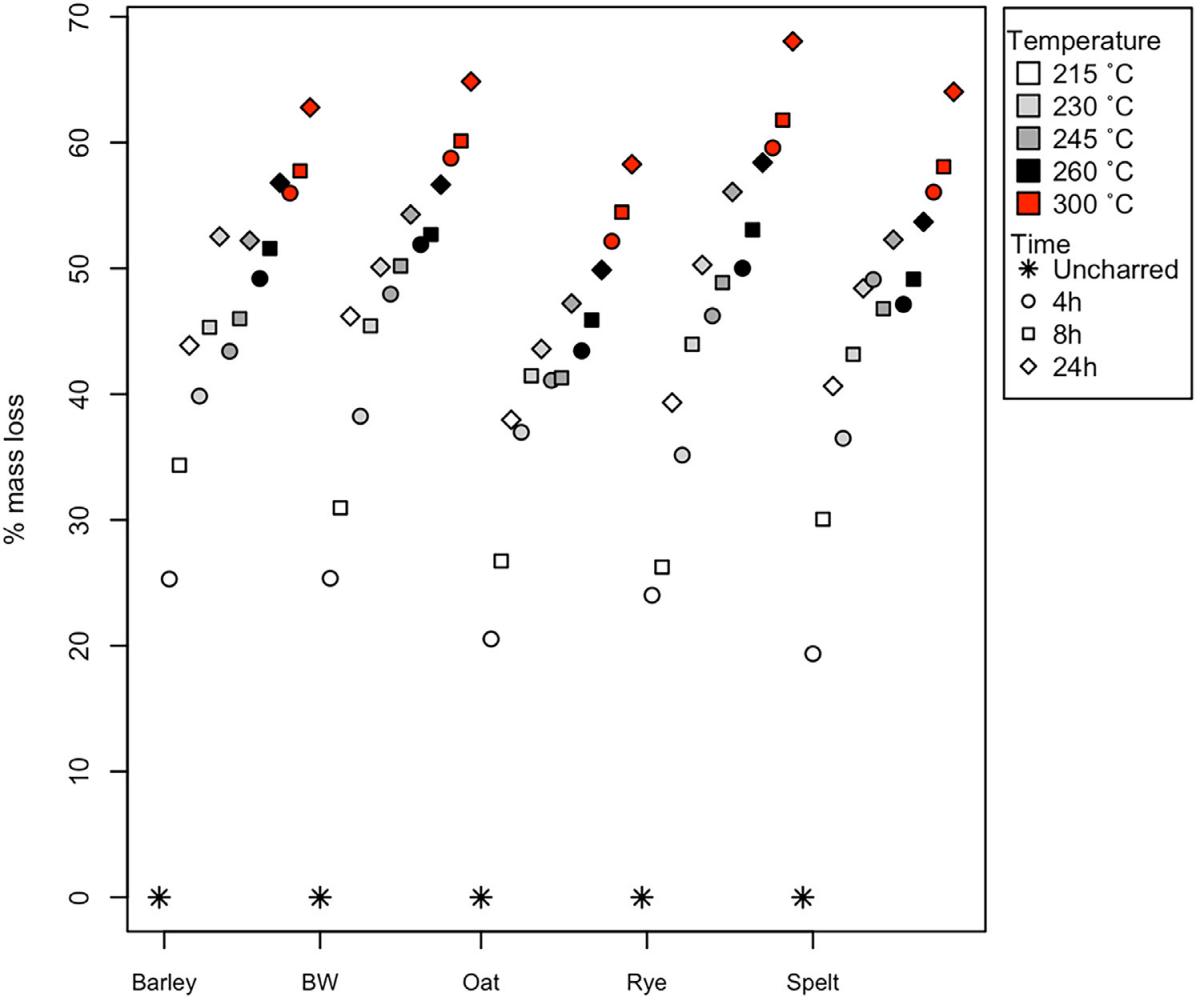
The averaged percentage mass loss of the three replicates for the different time and temperature charring combinations of species (hulled barley, bread wheat, oat, rye and spelt) charred up to 300 °C.

Dataset 1 contains the results of the stable carbon and nitrogen isotope analysis of the experimentally charred grains (primary and secondary data), including the raw and calibrated isotopic data as well as the %C and %N of the samples (also graphically represented in Fig. 1, Fig. 2 showing only species with 300 °C data). The standard uncertainty of each sample as calculated using the Kragten [4] approximation method is also included in Dataset 1, in the columns *δ*^15^N_AIR_ ± 1σ and *δ*^13^C_VPBD_ ± 1σ. Table 6 provides the fresh weight, charred weight and percent weight loss of the new samples, and bread wheat and barley samples from Nitsch et al. [2], and the mass loss for those species is graphically represented in Fig. 3.

Dataset 2 provides internal and external photographs of each species at each heating duration and temperature combination. The images include photos previously taken during the Nitsch et al. [2] experiment, while the rye, spelt, oat, and wheat (300 °C) and barley (300 °C) images are of newly charred material.

This publication also provides the link to the storage location of the raw data csv files and R script required for calculating the precision, accuracy and uncertainty shown in Table 5, as well as the regression models used in Stroud et al. [1]. This allows users to construct their own charring offset for their range of crop taxa. The R script also provides the code used to construct the figures in this publication as well as Stroud et al. [1].

## 4 Experimental Design, Materials and Methods

The data presented in this paper derive from the new experimental heating of the grains of five cereal species at 16 different time and temperature combinations. The aim was to understand the impact of heating both isotopically and morphologically on wheat, barley, rye, oat and spelt. This would allow for the calculation of a charring offset suitable for archaeobotanical assemblages containing such species, as well as the selection of suitable grains for isotopic analysis (see [1]). The methodology used for the experiment follows that of Nitsch et al. [2] so as to make it comparable with the isotopic data from their heating experiment on wheat and barley.

Nitsch et al. [2] published the stable carbon and nitrogen isotopic values of six different species which had been experimentally charred at 215 °C, 230 °C, 245 °C and 260 °C for 4 h, 8 h and 24 h. Nitsch et al. [2] also included data from dehusked hulled barley grains to understand the impact of the hull on the *δ*^13^C and *δ*^15^N values of grains (for full details see [2]). Note that images of these samples were not available from Nitsch et al. [2] and only isotopic data is available (see Dataset 1, hulled barley experiment 2 and pearled barley experiment 2). The raw data from the Nitsch et al. [2] experiments was obtained from data stores at the School of Archaeology, University of Oxford and was incorporated within the datasets presented here. The bread wheat and hulled barley data from Nitsch et al. [2] was combined with the new data produced for Stroud et al. [1]. The new dataset added an extra temperature to Nitsch et al. [2] set of four temperatures, with the addition of the heating temperature of 300 °C. The original raw data from the Nitsch et al. [2] publication, including all available raw isotopic data for their species was included in Dataset 1.

### 4.1 Materials

The Nitsch et al. [2] data derived from single growing conditions from the same farm in the same year (see Table 1 in [2]). No chemical fertilizers were used and the crop received no manure.

New data were obtained using rye, oat and spelt grains all grown on organic farms. Whitehall Farm in Peterborough (UK) provided the rye grains, Tamarisk Farm in Dorset (UK) provided the oat grains and the spelt grains came from experimental plot 26 at Sutton Bonnington (UK). The grains used all came from single fields and a single year. The experiment used the same hulled barely material used by Nitsch et al. [2], still located at the School of Archaeology, University of Oxford: a single field in the Sault region of Provence in France (code CHA-11). The bread wheat came from plot 18 of the Bad Lauchstädt long-term static fertilization experiment in Germany, the same location from which the Nitsch et al. [2] material was derived, however the newly charred material came from the harvest of 2004. This had, however, been grown under the same cultivation conditions as the Nitsch et al. [2] samples.

Eight hundred grains of rye, oat and spelt each were used, providing enough material to cover the 16 different combinations of temperature and time, including an unheated batch. A total 50 grains were selected for each of the heating combinations so as to provide enough material for both isotopic analysis and photography to assess the morphological changes of the grains. Three replicates of ten grains were used per heating condition, with the remaining 20 grains used for the photography. Each batch's weight was taken before and after heating to understand mass loss for each heating condition.

A similar method was followed for the new barley and wheat grains, with 200 grains used per species. This allowed a total of 50 grains to be selected for each of the new heating conditions (300 °C at 4 h, 8 h, and 24 h) as well as a batch left uncharred so as to compare comparability between the new data and old data.

### 4.2 Heating

A Gallenkamp Plus II electric oven at the School of Archaeology, University of Oxford was used for the experimental heating. Spelt, oat and rye were charred at five different temperatures for three different durations: 215 °C, 230 °C, 245 °C, 260 °C and 300 °C at 4 h, 8 h or 24 h. Furthermore, the new wheat and barley grains were charred for 4 h, 8 h or 24 h at 300 °C so as to extend the already existing Nitsch et al. [2] dataset to 300 °C. Following the same protocol as Nitsch et al. [2] the grains were wrapped in aluminium foil envelopes and buried in sand within beakers. The oven was preheated to the required temperature before the beakers were placed inside. The temperature of the oven was recorded throughout the duration of the heating using four thermocouples connected to a datalogger. Three of the thermocouples were buried inside beakers of sand at three points in the oven to understand the temperatures experienced by the grains, while a fourth thermocouple was placed in the oven to monitor the overall oven temperature outside the sand. Data shows that once the oven reached temperature, variability was less than 3%. Once the allotted heating time was reached the grains were removed from the oven and left to cool in the sand until they reached room temperature.

### 4.3 Isotopic analysis

Three replicates of the 16 temperature/duration combinations, a total of 48 samples per species, were isotopically analysed at the University of Oxford's Research Laboratory for Archaeology and the History of Art. Each replicate contained 10 grains which were homogenised using an agate mortar and pestle into a powder. As the uncharred material was too hard to homogenise by hand, it was homogenised using a Spex 2760 Freezer/Mill. The homogenised powders were weighed into tins for isotopic analysis, with one isotopic sample per replicate. The samples were analysed on a Sercon 20-22 EA-GSL isotope ratio mass spectrometer operating in continuous flow. Due to the variability of the %N of the different species, the samples had their nitrogen and carbon values measured in separate runs. The raw and drift corrected isotope ratios were calculated through comparison with an internal alanine standard.

A two-point calibration to convert the raw *δ*^13^C values to *δ*^13^C_VPDB_ was conducted using two bracketing reference materials: IAEA-C7 and IAEA-C6. A two-point calibration using IAEA- N1 and IAEA-N2 converted the raw *δ*^15^N values to *δ*^15^N_AIR_ for the majority of samples. A small number of samples which were re-run due to low nitrogen yields, were normalised using the internal standard of SEAL and EMA-P2 (runfile 200827). Data precision, accuracy and overall uncertainly were examined following Szpak et al. [3]. Check standards of Alanine, and EMA-P2 (when not used as a calibration standard) or Leucine were included in each run. Every tenth sample was duplicated and, following Szpak et al. [3], they were used in conjunction with the calibration standards, to understand accuracy and precision (see Tables 1, 2, 3, 4 and 5). Precision, or within laboratory random error was calculated as the root sum-square of the pooled standard deviations of all repeated measurements (check and calibrations standards and duplicates). Accuracy, or systematic measurement error, was calculated as the root-sum-square of the root-mean-square of the difference between the observed mean and the known value of check standards and the root-mean-square of the known standard deviation of the check standards [3]. The standard uncertainty of a given sample was calculated as the root-sum-square of the precision and accuracy. Table 5 shows the results of such calculations.

The standard uncertainty of each isotope value was also calculated following the Kragten approximation method [4]. This allowed measurement uncertainty of the new isotope samples and of the Nitsch et al. [2] data to be compared. The Nitsch et al. [2] dataset used the Kragten [4] method to understand measurement uncertainty and did not contain enough standards/replicates to allow for the Szpak et al. [3] method to be applied. Nitsch et al. [2] did not duplicate any samples and used slightly different calibration standards, with USGS40 used instead of IAEA-N2 (see [2]; supplementary table 1), as well as IAEA-N1 and IAEA-C7 and IAEA-C6. By calculating the standard uncertainty of the new data using the Kragten method, the overall average uncertainty of all data, new and old, could be calculated (see Table 5).

All graphing and statistical analysis was conducted using R-Studio, with R version 4.1.

### 4.4 Sectioning and Photography

The remaining 20 grains from each of the 16 different heating conditions were examined under a Lecia stereo microscope at 4× -16× magnification to understand changes to the morphology of the grains. Detailed photos using the Lecia microscope with an attached Lumenera infinity 3–6 UR camera were taken of the external morphology of a grain best representing the average distortion seen within each heating condition. Grains were sectioned in half at right angles to the grain's ventral groove allowing the internal structure of the grains to be examined under the microscope. The Lumenera infinity software was used to take the photos and add scale bars.

## Ethics Statement

This article does not contain any studies with human or animals subjects. The datasets used in the article are open to the public. For the usage of these datasets, proper citation rules should be maintained.

## CRediT authorship contribution statement

**Elizabeth Stroud:** Conceptualization, Methodology, Formal analysis, Investigation, Data curation, Visualization, Writing – original draft, Writing – review & editing. **Michael Charles:** Methodology, Visualization, Writing – review & editing. **Amy Bogaard:** Writing – review & editing, Supervision. **Erika Nitsch:** Resources. **Helena Hamerow:** Funding acquisition.

## Data Availability

Data files for Data in Brief charring paper (Original data) (Zenodo)
